# A Treatise on the Practice of Medicine

**Published:** 1861-09

**Authors:** 


ART. VI.—*A Treatise on the Practice of Medicine.* By EDWIN R. MAXSON, M.D., formerly Lecturer on the Institutes and Practice of Medicine in the Geneva Medical College. 8vo., pp. 705. Philadelphia: Lindsay & Blakiston, 1861.

THIS volume contains a compendious view of the whole field of medicine, including Pathology and Therapeutics, with a "glance at Anatomy and Physiology." It resembles, in its wide scope, the similar works of Barlow, Elliottson, and Watson, from the other side of the Atlantic; of Eberle, Dunglison, Wood, Dewees, and Dickson, of our own country. It might seem that the student and young practitioner were well supplied with such elementary books, but there is always room everywhere for anything that is good of its class—and all such essays must be judged upon their own particular merits.

At the very threshold we meet with the presentment of a special claim upon our attention:—

"I have attempted," the author tells us in his Preface, "to draw up the work without even the shadow of empiricism, by taking the human system in health as the standard; and then noting the deviations from that standard, constituting the various morbid conditions or diseases. By taking this course I have been enabled to arrive at clear indications of treatment, from direct pathological conditions, for every prescription which I have made."

We are compelled to say that we have not found these lofty pretensions sustained by the speculations or reasonings and methods of treatment proposed in the succeeding pages. We are persuaded that our divine science, with all its admirable progress, has not yet prevailed so far as to enable us to deduce "clear indications of treatment" and precise selection of "prescriptions," from any knowledge of "the various morbid conditions" of the body or its parts. We fear that the truth is still to be found in the modest disclaimer of Frerichs, one of the most profound of modern scientific inquirers, when he says: "Scientific medicine, although it has not rendered a rational system of treatment possible, has furnished us with important data, in anticipation of such a system. Between the scientific department of clinical medicine and that of real practice, there exists a chasm, which is bridged over, in a doubtful manner, at a few places only." We think our author will speak with somewhat less confidence on this topic of "clear indications" when he is a few years older.

An extensive practice has furnished him with much interesting material, and the reader will find many facts and observations in his histories of particular diseases worthy of record. It is chiefly in his theoretical views that he departs from the notions generally entertained among physicians, and we shall occupy a little space in commenting upon these views.

In his opening chapters upon the nature, origin, and causes of disease, he refers us directly to the fall of man as the sole source of suffering and death: "It is unreasonable to suppose that accidents, or even the elements of nature, which now appear to produce disease, would have harmed him, if he had retained his original purity." Is he aware that disease and death are known to have entered the world before the era of man's creation? Zeis, Wyman, and Agassiz tell us of fossil remains of that remote antiquity, exhibiting a variety of abnormal and "morbid conditions." Again, he declares that "it is absurd to suppose that God made contagions;" but in Chapter XIII., sections 9 and 10, he treats of "animalcular eruptions" and "cryptogamous eruptions." If the animalculæ and cryptogams causative of these "morbid conditions" were not created, they must be of spontaneous or equivocal generation—a heresy not tolerated for a moment by the theological school to which our author belongs. We leave him to select for himself the easiest horn of the dilemma.

Believing that human life, in the primal epoch, reached one thousand years, he ascribes the progressive shortening of its duration down to the present time, to the accumulative influence of successive violations of the laws of God and nature. He lays a peculiar stress, among these, upon "Irregularity in taking food," to which, apart from its quality and quantity, he ascribes, directly and indirectly, almost all "the ills that flesh is heir to." Page 25:—

> "If food be taken at irregular hours, or between meals, the gastro-intestinal mucous membrane becomes irritable; the nervous system becomes irritable; all the fluids and solid structures of the body become more or less deranged, and the patient becomes strongly predisposed to scrofula, consumption, dropsy, epilepsy, catalepsy, and, in fact, almost every variety of disease to which the human system is liable."

Tobacco, also, he dwells upon as "a fearful cause of disease, containing, as it does, three deadly poisonous principles, nicotia, nicotianin, and an empyreumatic oil;" and unhesitatingly sets it down as producing "mania a potu or delirium tremens." Page 298.

Of the nature of malaria, or Koino-miasmata, he speaks with unusual reserve, and proceeds "to raise the question, how far, if at all, Koino-miasmata would have been injurious to the human system, provided the laws of health had always been observed and our constitutions had never been depraved." In the latter case, he "inclines to the opinion that it would either have remained harmless to us, or else we should have retained good sense enough to have kept beyond its reach." Ignoring all the known differences of degree of liability to suffer from it among the various races of men, he espouses the doctrine of acclimatation, admitting not only that "the system gradually becomes accustomed to its influence, but it is said even requires it;" a rather ultra view of the matter, for which we know not his authority.

He seems somewhat embarrassed, theologically, with the origin and spread of epidemics, but "inclines to the opinion" that those are wrong who "have placed the cause in some mysterious principle beyond the power of man to reach or modify, as though the Almighty had placed us here, and let loose upon us occasional emissaries of destruction, without our own fault, or even ability to palliate or resist."

He seems to agree with Forbes, that as "medicine is, at best, little better than a necessary evil," "nothing should be prescribed as a remedy without first getting a clear indication." In all doubtful cases, therefore, the expectant method is to be followed; but, as we intimated above, he differs greatly from Forbes, and all other such skeptics, as to the readiness with which "a clear indication" is attainable in the present state of our knowledge. Thus, under the head of "Intussusception," which he treats of, very originally, as a special malady, he says, "Such are plainly the indications of this obscure and truly dangerous affection." Dividing the attacks into "direct and retrograde," he manages them with a logical reciprocity; if produced or preceded by catharsis, he administers emetics; if by vomiting, he exhibits cathartics.

We find ourselves carried back into genuine Broussaisism on page 72, where such a confusion is established between general fever and gastro-enteritis "as to make it a matter of doubt which is the cause and which is the effect." Yet he pronounces that "intermittent fever is essentially a disease of the nervous system," and "seriously questions whether it should be regarded as a fever, or as an intermittent neuralgia." He considers it proved that "gastro-enteritis, together with the influence of the paludal poison, is the cause of bilious-remittent fever," thus solving the difficult question as to the relation between remittents and intermittents. In the treatment of the latter, he adds iron-wood to the long list of remedies, and does not hesitate to administer quinine in remittents, and is "persuaded, from extensive and careful observation, that its effects are most salutary," although "it may appear strange to some that quinine should be given in two or three grain doses, every six hours, when there is a high state of febrile excitement." Page 109.

Like some others of our Western brethren, he admits and treats of a "Simple Continued Fever," distinct from the enteric or typhoid. He devotes to this subject an entire section of his Chapter IV., "On General Fevers," which is worthy of, and will repay an attentive perusal. He places diphtheria under this same category, in another section, as an idiopathic fever.

In a notice of the special views entertained by the author, it would be improper to omit his remarks on ablution, page 605:—

"Too much washing and fretting the skin increases its filth, by calling to the surface fluids that ought to be thrown off by the kidneys and bowels, and is very apt to produce constipation. If we had been designed by the Creator for aquatic animals, we should have been supplied with fins instead of legs."

The common opinion that the drinking of water impregnated with lime produces or increases a liability to stone in the bladder, has, we know, been opposed by many authorities, among them the distinguished pathologist and professor, editor of this journal; but we have not before met with the seemingly paradoxical notion that such waters are corrective and protective, as is maintained by Dr. Maxson, who says, page 648:—

"It appears probable, that the constant use of lime, as it exists in the water in limestone regions, tends strongly to prevent a lithic acid diathesis and deposit, and hence is a partial security against the formation of urinary calculi of all kinds."

The style of the treatise is plain, simple, and intelligible, but often inelegant. The book, which is otherwise well got up, is defaced by an unusual number of typographical errors, among which we suppose we must include the phrase "incontinuance of urine," which occurs nine times on one page.

